# Association of volumetric-modulated arc therapy with radiation pneumonitis in thoracic esophageal cancer

**DOI:** 10.1093/jrr/rrac021

**Published:** 2022-05-20

**Authors:** Hiroyuki Inoo, Katsuyuki Sakanaka, Kota Fujii, Yuichi Ishida, Takashi Mizowaki

**Affiliations:** Department of Radiation Oncology and Image-Applied Therapy, Graduate School of Medicine, Kyoto University, 54 Shogoin Kawahara-cho, Sakyo-ku, Kyoto, 606-8507, Japan; Department of Radiation Oncology and Image-Applied Therapy, Graduate School of Medicine, Kyoto University, 54 Shogoin Kawahara-cho, Sakyo-ku, Kyoto, 606-8507, Japan; Department of Radiation Oncology and Image-Applied Therapy, Graduate School of Medicine, Kyoto University, 54 Shogoin Kawahara-cho, Sakyo-ku, Kyoto, 606-8507, Japan; Department of Radiation Oncology and Image-Applied Therapy, Graduate School of Medicine, Kyoto University, 54 Shogoin Kawahara-cho, Sakyo-ku, Kyoto, 606-8507, Japan; Department of Radiation Oncology and Image-Applied Therapy, Graduate School of Medicine, Kyoto University, 54 Shogoin Kawahara-cho, Sakyo-ku, Kyoto, 606-8507, Japan

**Keywords:** esophageal cancer, radiotherapy, radiation pneumonitis (RP), volumetric-modulated arc radiotherapy (VMAT)

## Abstract

The lung volume receiving low-dose irradiation has been reported to increase in volumetric-modulated arc radiotherapy (VMAT) compared with three-dimensional conformal radiotherapy (3DCRT) for thoracic esophageal cancer, which raises concerns regarding radiation pneumonitis (RP) risk. This single institutional retrospective cohort study aimed to explore whether VMAT for thoracic esophageal cancer was associated with RP. Our study included 161 patients with thoracic esophageal cancer, of whom 142 were definitively treated with 3DCRT and 39 were treated with VMAT between 2008 and 2018. Radiotherapy details, dose–volume metrics, reported RP risk factors and RP incidence were collected. The RP risk factors were assessed via multivariate analysis. Dose–volume analysis showed that VMAT delivered more conformal dose distributions to the target volume (*P* < 0.001) and reduced V_30 Gy_ of heart (57% vs 41%, *P* < 0.001) but increased V_5 Gy_ (54% vs 41%, *P* < 0.001) and V_20 Gy_ (20% vs 17%, *P* = 0.01) of lungs compared with 3DCRT. However, the 1-year incidence rates of RP did not differ between the two techniques (11.3% in 3DCRT vs 7.7% in VMAT, *P* = 0.53). The multivariate analysis suggested that the presence of interstitial lung disease (ILD) (*P* = 0.01) and V_20 Gy_ of lungs ≥20% (*P* = 0.008) were associated with RP. Conclusively, VMAT increased the lung volume receiving low to middle doses irradiation, although this might not be associated with RP. Further studies are needed to investigate the effect of using VMAT for delivering conformal dose distributions on RP.

## INTRODUCTION

Radiotherapy is an established definitive treatment for thoracic esophageal cancer [1, 2]. One of the most critical adverse events of radiotherapy in treating esophageal cancer is radiation pneumonitis (RP). Acute RP occurs 2–6 months after radiotherapy, and it is followed by radiation-induced lung fibrosis within 12 months [3, 4]. The incidence rates of this lung injury have been reported to be between 4% and 35% [5–7]. Risk factors of RP after radiotherapy for esophageal cancer include age, smoking history, the tumor volume, the mean lung dose, the lung volume receiving 20 Gy (V_20 Gy_) and the lung volume receiving low-dose irradiation [5, 8–11].

Intensity-modulated radiation therapy (IMRT) is an advanced radiotherapy technique compared with three-dimensional conformal radiotherapy (3DCRT). The benefits of IMRT are that it improves target conformity, reduces doses to heart and coronary artery and reduces the lung volume irradiated high dose compared with 3DCRT method in thoracic esophageal cancer [12, 13]. In contrast, one of the demerits of IMRT is that it increases the volume of normal tissues receiving low-dose irradiation surrounding the target [12, 13]. IMRT utilizes multiple irradiation fields or arcs that pass through bilateral lungs before reaching the target that is located in the mediastinum. This method potentially increases the volume receiving low-dose irradiation bilateral lungs that surround the primary esophageal tumor and the metastatic lymph nodes compared with 3DCRT, which, subsequently, might increase the incidence of RP. Therefore, IMRT may not always be the optimal technique for thoracic esophageal cancer, especially with respect to the lung doses.

Retrospective cohort studies have suggested that the use of IMRT for esophageal cancer could reduce the incidence or severity of RP compared with that of 3DCRT [11, 14–17]. However, these studies have several drawbacks. For instance, some reports failed to include any information on the lung dose–volume metrics of 3DCRT and IMRT [16, 17]. In other reports, the researchers irradiated unacceptably large lung volume in 3DCRT, that the lung volume receiving 5 Gy were 75.5–79.7%, and have not provide any details pertaining to the arrangement of their fields [11, 15]. Furthermore, inconsistent results have been published regarding the lung volume receiving low-dose irradiation in IMRT. One study has previously reported that IMRT could reduce the lung volume receiving low to middle doses irradiation compared with 3DCRT [11], whereas other studies concluded the opposite [14, 15]. Consequently, the precise effect of IMRT on the lung dose and the risk incidence of RP have yet to be fully established in thoracic esophageal cancer.

The aims of the current study were to retrospectively compare the dose–volume indices of patients treated with four-field 3DCRT using anterior–posterior-opposed and oblique-opposed fields (4F-3DCRT) to that of patients treated with volumetric-modulated arc therapy using the split arc technique (split arc [SA]-volumetric-modulated arc radiotherapy [VMAT]) in thoracic esophageal cancer using the single institutional database and to explore the association between the radiation technique used and the development of RP.

## MATERIALS AND METHODS

The current study was a single institutional retrospective cohort study. The Institutional Review Board of the Kyoto University Graduate School and Faculty of Medicine, Ethics Committee approved the current study on March 16, 2017 (R1048). The study was conducted in accordance with the Declaration of Helsinki and the Japanese Ethical Guidelines for Epidemiological Research.

### Inclusion criteria

**Fig. 1:**
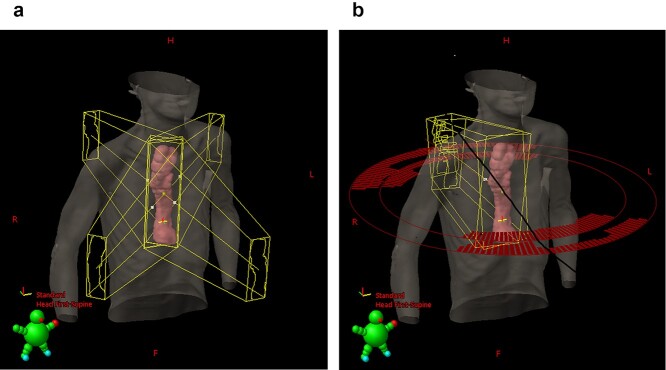
Beam arrangements of; (a) four-field three-dimensional conformal radiotherapy using anterior–posterior-opposed and oblique-opposed fields; and (b) volumetric-modulated arc therapy using split arc technique.

Inclusion criteria for the current study were as follows: patients who underwent radiotherapy at our hospital from April 2008 to March 2018 and were histologically diagnosed with thoracic esophageal cancer; patients with clinical stages I–IV according to the Union for International Cancer Control (UICC) TNM Classification of Malignant Tumors, eighth edition; patients without distant organ metastasis and previous therapy for esophageal cancer; 4F-3DCRT or SA-VMAT was performed (Fig. 1); ≥50.4 Gy was prescribed to all gross tumors; and patients who had a follow-up of at least 1 year without developing RP or until the incidence of RP within 1 year from the final day of radiotherapy through physical examinations, laboratory tests and computed tomography (CT) findings.

### Collected information

The previously reported risk factors for RP were retrospectively collected: age [8]; smoking history [11]; the size of the planning target volume (PTV) [11], which have already been reported as risk factors for RP in esophageal cancer; gender [18]; Eastern Cooperative Oncology Group performance status [18]; use of concurrent chemotherapy [19]; and the presence of interstitial lung disease (ILD) [20], which has been reported as an essential risk factor for RP in lung cancer. Our study additionally collected the following information through medical records: histology, clinical stage based on UICC TNM Classification of Malignant Tumors, eighth edition; anatomical subsites; and details of radiotherapy, the use of elective nodal irradiation and the incidence of RP after chemoradiotherapy. The data of the current study were fixed on 9 August 2021.

### Delineation of irradiation targets and organs at risk

All patients underwent CT simulation in a supine position. The primary tumor and metastatic lymph nodes were contoured as gross tumor volumes with reference to contrast-enhanced CT, 18-fluorodeoxyglucose positron emission tomography and esophagogastroduodenoscopy. Target delineation was the same between 4F-3DCRT and SA-VMAT as described previously [21]. We created the clinical target volume (CTV) of the primary esophageal tumor with a 2-cm head-to-tail margin along the esophagus plus a 0- to 0.5-cm margin in the radial direction and the CTV of metastatic lymph nodes with a 0.5-cm margin to the nodes. The CTV of subclinical regional lymph nodes were defined according to the location of the primary tumor—the supraclavicular to superior mediastinal lymph nodes for an upper thoracic esophagus, the mediastinal to perigastric lymph nodes for a middle thoracic esophagus, and the mediastinal to celiac axis lymph nodes for a lower thoracic esophagus. The PTV were created by adding a 0.5- to 1-cm margin to the CTV of the primary esophageal tumor plus metastatic lymph nodes and the PTV of subclinical regional lymph nodes were created by adding a 0.5- to 1-cm margin to the CTV of subclinical regional lymph nodes, respectively. We did not use breath-hold method as respiratory motion countermeasures and treated under free-breath method. Non-helical CT and four-dimensional CT (4D-CT) were obtained using a 16-slice scanner. When non-helical CT images were obtained, the targets with a large amount of respiratory motion, for example abdominal lymph nodes, added larger PTV margin and when 4D-CT images were obtained monitoring the respiration with a real-time position management system on the patient’s abdomen, respiratory motion of targets were confirmed by average-intensity projection images generated considering an average of 10 respiratory cycle phases of the 4D-CT images. The spinal cord, heart and lungs were contoured as organs at risk. The delineation of heart followed JCOG0909, a phase II trial of definitive chemoradiotherapy for thoracic esophageal cancer, in the current study. It included the heart and ascending aorta from the level of brachiocephalic artery to the bottom of heart, and the pulmonary vasculature in mediastinum [22].

### Details of 4F-3DCRT and SA-VMAT

The included patients were treated with either 4F-3DCRT or SA-VMAT. Radiotherapy techniques were selected by radiation oncologists depending on the patients’ condition and disease. SA-VMAT was selected when the delivery of adequate doses by 4F-3DCRT was difficult due to gross tumors bilaterally located in mediastinum or supraclavicular lymph nodes. 4F-3DCRT used anterior–posterior-opposed and oblique-opposed fields (Fig. 1a). The dose was prescribed according to the International Commission on Radiation Units points of the PTVs in 1.8–2.0 Gy per fraction. Elective nodal irradiation was performed except for patients with clinical T1 tumor without metastatic lymph nodes or frail patients. Following 40–41.4 Gy of elective nodal irradiation, irradiation fields were cone downed to the PTV of the primary esophageal tumor and the metastatic lymph nodes. The gantry angles of the 4F-3DCRT oblique fields ranged between 135° and 160° and between 315° and 340°. The leaf margin of the 4F-3DCRT irradiation fields was set to 0.5 cm from the PTVs; however, this was reduced to <0.5 cm to minimize the spinal cord dose to <50 Gy.

SA-VMAT used split coplanar arcs to suppress the lung volume receiving low dose irradiation and prevent lateral beam entry from around 225° to 315° and from around 45° to 135° (Fig. 1b). Some patients underwent SA-VMAT by two-step method; 40 Gy was delivered to subclinical lymph nodes, and the irradiation fields were cone-downed to gross tumors after 40 Gy. Other patients underwent SA-VMAT by simultaneous-integrated boost method delivering 1.8–2.3 Gy per fraction to gross tumors, and the subclinical regional lymph nodes with 1.6–1.8 Gy per fraction. During optimization, we attempted to reduce the dose to heart and lungs by maintaining target coverage and homogeneity. Dose constraints for the spinal cord were defined as follows: maximum point dose <50 Gy. We performed image-guided radiotherapy for daily correction; stereotactic kV X-ray imaging was used in 4F-3DCRT, and cone beam CT and kV X-ray imaging were used in SA-VMAT, respectively.

### Dose–volume metrics of irradiation targets and organs at risk

The current study recalculated the dose–volume metrics of the PTV, heart and lungs in the included 4F-3DCRT and SA-VMAT plans using the same dose calculation algorithm: Acuros XB version 15.6.05 (Varian Medical Systems). For recalculation, we used the originally calculated monitor units for clinical uses in 4F-3DRT and SA-VMAT. For the evaluation of the conformity and homogeneity of the PTV, the current study used the conformity index, which is equal to the volume of the body receiving at least 95% of the prescribed dose (V_95%_) divided by the volume of the PTV (V_PTV_) and the homogeneity index (HI) = (D_2%_ - D_98%_)/D_50%_, where D_X%_ represent the doses received by X% and PTV volumes, according to the International Commission on Radiation Units and Measurements 83. We then calculated the percentage volume receiving 30 Gy (V_30 Gy_) of heart and the mean heart dose, V_5 Gy_, V_10 Gy_, V_20 Gy_ and V_30 Gy_ of lungs and the mean lung dose in both 4F-3DCRT and SA-VMAT.

### Follow-up after radiotherapy and diagnosis of radiation pneumonitis

All included patients were followed up within a 3-month period following radiotherapy, and their condition was assessed with physical examinations, laboratory tests and CT scans. Two radiation oncologists (H.I. and K.S.) reviewed the medical records and CT images of the included patients and retrospectively diagnosed and graded the RP following the Common Terminology Criteria for Adverse Events version 5.0. Diagnosis of RP was based on symptoms, laboratory data and imaging studies. Respiratory symptoms with ground-glass opacity or consolidative changes in the radiation fields treated with steroid medications and antibiotics were considered typical symptoms that led to the diagnosis of RP. Consequently, the number of patients who developed RP up until 12 months after the final day of radiotherapy for esophageal cancer was measured.

### Statistical analysis

Differences of categorical and continuous variables of patient characteristics and dose–volume metrics between 4F-3DCRT and SA-VMAT were examined using Fisher’s exact test and Mann–Whitney U test, respectively. The scatter plot, composed of V_20 Gy_ of lungs in the horizontal axis and V_30 Gy_ of heart in the vertical axis, was created to show associations of dose volume metrics of heart and lungs and the incidence of RP depending on the esophageal primary site and radiotherapy techniques. The risk factors for ≥grade 2 RP were examined using the Cox proportional hazard model as a univariate analysis. The included risk factors were dose–volume metrics of lungs [5, 9, 10], age [8], smoking history [11], size of the PTV [11], gender [18], performance status [18], use of concurrent chemotherapy [19], presence of ILD [20] and radiotherapy techniques. V_5 Gy_ ≤ 50% and V_20 Gy_ ≤ 20% were then selected as the cutoff values for the irradiated lung volumes in accordance with the National Comprehensive Cancer Network guidelines [1]. The identification of confounding factors between the radiotherapy techniques and the RP risk factors with a *P*-value <0.05 led us to perform an adjusted factor analysis using the Cox proportional hazard model to evaluate their impact on RP. Furthermore, variables that reached *P*-value <0.05 in the univariate analysis, while adding these confounding factors to the reported factors, were further evaluated by the multivariate analysis using the Cox proportional hazard model.

All statistical analyses were performed with EZR, version 1.53 (Saitama Medical Center, Jichi Medical University, Saitama, Japan) [23], which is a graphical user interface for R (The R Foundation for Statistical Computing, Vienna, Austria). More precisely, it is a modified version of the R commander designed to add statistical functions that are frequently used in biostatistics.

## RESULTS

### Patient cohort

Two hundred ninety-six patients with clinical stages I–IV, histologically diagnosed thoracic esophageal cancer without distant organ metastasis, and previous treatment for esophageal cancer underwent radiotherapy of ≥50.4 Gy at our hospital from April 2008 to March 2018. Forty-two patients were excluded from the analysis because they underwent different beam arrangements from those used in 4F-3DCRT or SA-VMAT. In addition, 73 patients were excluded from the analysis because of having <1-year follow-up period or no follow-up CT after radiotherapy. Consequently, 181 patients met the inclusion criteria, of whom 142 underwent 4F-3DCRT and 39 underwent SA-VMAT.

Characteristics of patients who underwent 4F-3DCRT and SA-VMAT are summarized in Table 1. The median prescribed dose was 60 Gy (range, 50.4–66 Gy in 4F-3DCRT and 50.4–69 Gy in SA-VMAT) in both modalities (see Supplementary Table 1 for details of the prescribed dose). Two patients underwent SA-VMAT using two-step method, and 28 patients underwent SA-VMAT using simultaneous-integrated boost method. The representative of dose fractionation of simultaneous-integrated boost method as follows; the prescribed doses were 50.4 Gy in 1.8 Gy per fraction for PTVs with 44.8 Gy in 1.6 Gy per fraction for elective lymph nodal area, 60 Gy in 2 Gy per fraction for PTVs with 48 Gy in 1.6 Gy per fraction for elective lymph nodal area, respectively. Seven patients were diagnosed as ILD and did not show any respiratory symptoms. ILD was first noted accidentally in six patients during examinations for esophageal cancer. SA-VMAT was frequently used for upper thoracic esophageal cancer (*P* < 0.001), advanced clinical stage (*P* = 0.009) and large PTV (*P* < 0.001). Following comparisons between 4F-3DCRT and SA-VMAT, these were considered confounding factors for RP, and the stratification analysis was performed using these factors.

**Table 1:** Patient characteristics

	Four-field three-dimensional conformal radiotherapy (*n* = 142)	Split arc volumetric-modulated arc therapy (*n* = 39)	*P*-value
Gender (male/female)	112/30	32/7	0.82
Median age (years) (range)	68 (36–86)	70 (44–83)	0.28
Eastern Cooperative Oncology Group performance status (0/≥1)	103/39	34/5	0.06
Upper thoracic esophagus/middle to lower thoracic esophagus	11/131	20/19	<0.001
Histology (squamous cell carcinoma/others)	138/4	39/0	0.58
Clinical stage^*^ (I–III/IV)	93/49	16/23	0.009
Smoking history (smoker/non-smoker)	115/27	32/7	1
Use of concurrent chemotherapy (Yes/No) ^†^	135/7	38/1	1
Presence of interstitial lung disease (Yes/No)	5/137	2/37	0.65
Median planning target volume (cc) (range)	144 (41–560)	245 (89–425)	<0.001
Median prescribed dose to the planning target volume (Gy) (range)	60 (50.4–66)	60 (50.4–69)	0.12
Elective nodal irradiation (Yes/No)	104/38	30/9	0.84
Median V_5 Gy_ of lungs (%) (range)	40.9 (5.4–70.1)	54.4 (18.9–70.5)	<0.001
Median V_10 Gy_ of lungs (%) (range)	31.6 (3.9–50.2)	36.4 (11.2–48.2)	<0.001
Median V_20 Gy_ of lungs (%) (range)	16.5 (1.9–31.7)	19.5 (4.2–30.7)	0.01
Median V_30 Gy_ of lungs (%) (range)	7.7 (0.5–19.2)	9.2 (1.3–20)	0.32
Median value of mean lung dose (Gy) (range)	9.1 (1.3–15.5)	10.9 (3.9–16.3)	<0.001
Median V_30 Gy_ of heart (%) (range)	56.5 (45.5–81.6)	40.8 (22.3–73.2)	<0.001
Median value of mean heart dose (Gy) (range)	30.4 (0.6–45)	28.8 (0.2–40.3)	0.02
Conformity index^‡^	2.7 (1.3–6.5)	1.3 (1.0–2.8)	<0.001
Homogeneity index^§^	0.10 (0.05–0.54)	0.13 (0.06–0.18)	0.19

**Note:**  ^*^Clinical stage was based on the UICC TNM Classification of Malignant Tumors, eighth edition. ^†^Concurrent chemotherapy regimens were as follows: cisplatin plus 5-fluorouracil in 142 patients, nedaplatin plus 5-fluorouracil in six patients, 5-fluorouracil alone in 22 patients and cisplatin alone in three patients. ^‡^The volume receiving 95% dose/the volume of the PTV. ^§^(The dose receiving 2% of the PTV —the dose receiving 98% of the PTV)/the dose receiving 50% of the PTV.

**Abbreviations:** V_X Gy_; the percentage of target volume receiving X Gy.

### Comparison of dose–volume metrics between 4F-3DCRT and SA-VMAT

**Fig. 2:**
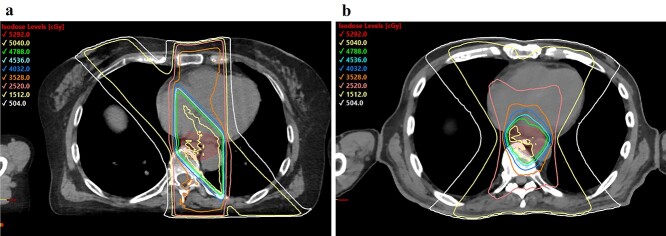
Representative isodose distributions of: (a) four-field three-dimensional conformal radiotherapy using anterior–posterior-opposed and oblique-opposed fields; and (b) volumetric-modulated arc therapy using split arc technique.

**Fig. 3:**
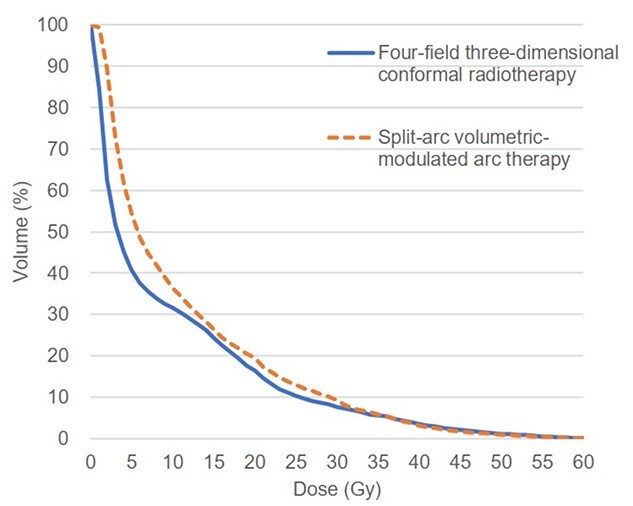
Median cumulative dose–volume histograms of lungs in patients treated with four-field three-dimensional conformal radiotherapy using anterior–posterior-opposed and oblique-opposed fields and volumetric-modulated arc therapy using split arc technique.

Representative dose distributions of patients who underwent 4F-3DCRT and SA-VMAT are presented in Fig. 2. SA-VMAT delivered more conformal doses to PTV than 4F-3DCRT while maintaining homogeneity. Furthermore, the conformity index was 1.3 (range, 1.0–2.8) in SA-VMAT, and 2.7 (range, 1.3–6.5) in 4F-3DCRT (*P* < 0.001). The homogeneity index was 0.13 (range, 0.06–0.18) in SA-VMAT and 0.10 (range, 0.05–0.54) in 4F-3DCRT (*P* = 0.19) (Table 1). Conformal dose delivery of SA-VMAT decreased the cardiac volumes receiving irradiation compared with that of 4F-3DCRT (Table 1); however, V_5 Gy_, V_10 Gy_ and V _20 Gy_ of lungs and the mean lung doses were increased in SA-VMAT than in 4F-3DCRT (Table 1 and Fig. 3). The mean increases of V_5 Gy_ and V_20 Gy_ of lungs in SA-VMAT than in 4F-3DCRT were 13.5% (*P* < 0.001) and 3% (*P* = 0.01), respectively.

PTV volume and V_5 Gy_ of lungs was larger in SA-VMAT than 4F-3DCRT regardless of the primary sites (Table 2). Mean lung dose and V_30 Gy_ of heart in middle to lower thoracic esophageal tumor showed significant difference between two techniques. They were compatible with whole cohort analysis. No significant difference was observed in the other factors which was not compatible with whole cohort analysis.

**Table 2:** Relationship between radiotherapy techniques and dose volume metrics for each location of esophageal primary tumor

	Upper thoracic esophagus			Middle to lower thoracic esophagus		
	Four-field three-dimensional conformal radiotherapy (*n* = 11)	Split arc volumetric-modulated arc therapy (*n* = 20)	*P*-value	Four-field three-dimensional conformal radiotherapy (*n* = 131)	Split arc volumetric-modulated arc therapy (*n* = 19)	*P*-value
Median PTV volume (cc)	131	243	0.002	145	266	<0.001
Median V_5 Gy_ of lungs (%)	39.1	53.8	0.014	41.2	54.4	<0.001
Median V_20 Gy_ of lungs (%)	17.3	20.3	0.104	16.3	18.1	0.104
Median value of mean lung dose (Gy)	9.4	11.1	0.095	9.1	10.7	0.015
Median V_30 Gy_ of heart (%)	42.8	25.7	0.54	57.6	44.7	0.037
Median value of mean heart dose (Gy)	22.7	22.2	0.95	30.7	29.7	0.65
Number of patients with ≥grade 2 radiation pneumonitis (percentage, %)	0 (0)	2 (10)	0.53	16 (12.2)	1 (5.3)	0.70

**Abbreviations:** V_X Gy_; the percentage of lung volume receiving X Gy.

### Incidence of radiation pneumonitis

Nineteen patients experienced ≥grade 2 RP. The median onset of ≥grade 2 RP was 4.6 months (range, 0.3–12.0) in 4F-3DCRT and 3.7 months (range, 3.6–8.5) in SA-VMAT. The 1-year incidence rates of ≥grade 2 RP were 11.3% (16/142) in patients treated with 4F-3DCRT (grade 2 in four patients; grade 3 in seven patients; grade 4 in two patients; and grade 5 in three patients) and 7.7% (3/39) in patients treated with SA-VMAT (grade 2 in two patients and grade 3 in one patient).

### Risk factors for radiation pneumonitis

**Fig. 4:**
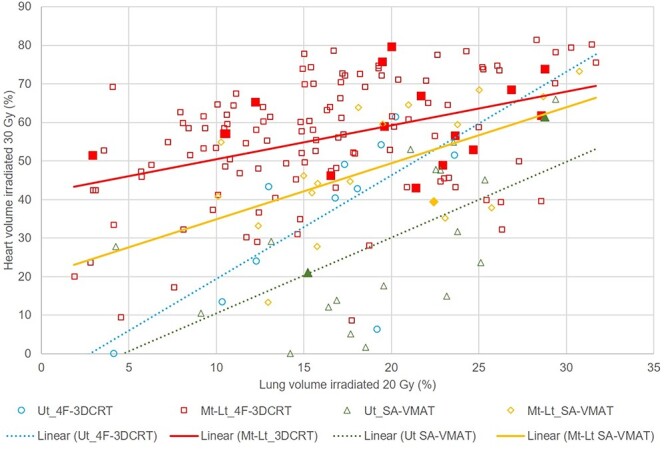
Scatter plot of V_30 Gy_ of heart and V_20 Gy_ of lungs depending on the locations of esophageal primary tumor and radiotherapy techniques. Abbreviations: 4F-3DCRT, four-field three-dimensional conformal radiotherapy; SA-VMAT, volumetric-modulated arc therapy using the split arc technique; Ut, upper thoracic esophageal tumor; Mt, middle thoracic esophageal tumor; Lt, lower thoracic esophageal tumor. Footnotes: blank shapes mean the patients without ≥ grade 2 RP, and filled shapes mean the patients with ≥ grade 2 RP.

The univariate analysis showed that the incidence of ≥grade 2 RP did not differ between the two radiotherapy techniques used in this study (11.3% vs 7.7%, *P* = 0.53). The stratified analysis of the three identified confounding factors did not reveal any differences regarding the 1-year incidence of ≥grade 2 RP between 4F-3DCRT and SA-VMAT: location of esophageal primary tumor (*P* = 0.75), clinical stage (*P* = 0.33) and the volume of PTV (*P* = 0.25). The incidence rate of RP did not show any significant difference between 4F-3DCRT and SA-VMAT regardless of primary tumor location (Table 2). The patients with the larger irradiated volume of heart and lungs tended to develop RP (Fig. 4). Then, the univariate analysis performed on other factors suggested that older age (*P* = 0.04), V_20 Gy_ > 20% (*P* = 0.02), and the presence of ILD (*P* = 0.009) were positively associated with the incidence of ≥grade 2 RP (Table 3). ILD was a risk factor for RP. Then we added the analysis using the data set of whole cohorts of the current study excluding patients with ILD (five patients in 4F-3DCRT and two patients in SA-VMAT). The incidence rate of RP was 10.2% in 4F-3DCRT and 5.4% in SA-VMAT, *P* = 0.39, respectively, which was consistent with the results from the analysis using the dataset including the patients with ILD.

**Table 3:** Incidence of RP

Variables		Number of patients	Number of patients with ≥grade 2 radiation pneumonitis (percentage, %)	Univariate analysis		Multivariate analysis	
				Hazard ratio (95% confidence interval)	*P*-value	Hazard ratio (95% confidence interval)	*P*-value
Gender	Female	37	2 (5.4)				
	Male	144	17 (11.8)	2.2 (0.52–9.7)	0.28		
Age	<70 years	99	6 (6.1)				
	≥70 years	82	13 (15.9)	2.7 (1.04–7.2)	0.04	2.7 (0.99–7.1)	0.052
Location of esophageal primary tumor	Upper thoracic esophagus	31	2 (6.5)				
	middle or lower thoracic esophagus	150	17 (11.3)	1.8 (0.42–7.8)	0.43		
Eastern Cooperative Oncology Group performance status	0	137	13 (9.5)				
	≥1	44	6 (13.6)	1.5 (0.57–3.9)	0.42		
Clinical stage^*^	I–III	109	8 (7.3)				
	IV	72	11 (15.3)	2.2 (0.89–5.5)	0.09		
Smoking history	No	34	2 (5.9)				
	Yes	147	17 (11.6)	2.0 (0.46–8.7)	0.35		
Use of concurrent chemotherapy	No	8	1 (12.5)				
	Yes	173	18 (10.4)	0.87 (0.12–6.5)	0.89		
Presence of interstitial lung disease	No	174	16 (9.2)				
	Yes	7	3 (42.9)	5.2 (1.50–17.8)	0.009	5.0 (1.4–17.6)	0.01
Planning target volume	<168 cc	90	6 (6.7)				
	≥168 cc	91	13 (14.3)	2.3 (0.86–6.0)	0.10		
V_5 Gy_ of lungs	≤50%	134	11 (8.2)				
	>50%	47	8 (17.0)	2.3 (0.91–5.6)	0.08		
V_20 Gy_ of lungs	≤20%	121	8 (6.4)				
	>20%	60	11 (18.3)	3.0 (1.19–7.3)	0.02	3.5 (1.4–8.6)	0.008
Radiotherapy technique	Four-field three-dimensional conformal radiotherapy	142	16 (11.3)				
	Split arc volumetric-modulated arc therapy	39	3 (7.7)	0.67 (0.20–2.3)	0.53		

**Note:**  ^*^Clinical stage was based on the UICC TNM Classification of Malignant Tumors, eighth edition.

**Abbreviations:** V_X Gy_; the percentage of the target volume receiving X Gy.

The multivariate analysis suggested that the presence of ILD (hazard ratio = 5.0 [95% confidence interval, 1.4–17.6], *P* = 0.01) and V_20 Gy_ of lungs >20% (hazard ratio = 3.5 [95% confidence interval, 1.4–8.6], *P* = 0.008) were associated with the incidence of ≥grade 2 RP (Table 3).

The association between the radiation techniques used and the dose–volume thresholds with RP is summarized in Table 4. The hazard ratio for the development of ≥grade 2 RP in SA-VAMT was smaller than in 4F-3DCRT for V_5 Gy_ of lungs >50% (1.3 and 3.6) and V_20 Gy_ of lungs >20% (2.5 and 3.3). V_5 Gy_ of lungs >50% (*P* = 0.01) and V_20 Gy_ of lungs >20% (*P* = 0.02) were associated with the development of ≥grade 2 RP in 4F-3DCRT, as opposed to those in SA-VMAT (*P* = 0.83 and 0.46, respectively).

**Table 4:** Association of radiotherapy techniques and dose–volume thresholds of lungs with RP

		Number of patients	Number of patients with ≥grade 2 radiation pneumonitis (percentage, %)	Hazard ratio (95% confidence interval)	*P*-value
Four-field three-dimensional conformal radiotherapy	V _5 Gy_ ≤ 50%	119	10 (8.4)		
	V _5 Gy_ > 50%	23	6 (26.1)	3.6 (1.31–9.9)	0.01
Split arc volumetric-modulated arc therapy	V _5 Gy_ ≤ 50%	15	1 (6.7)		
	V _5 Gy_ > 50%	24	2 (8.3)	1.3 (0.12–14.4)	0.83
All modality	V _5 Gy_ ≤ 50%	134	11 (8.2)		
	V _5 Gy_ > 50%	47	8 (17)	2.3 (0.91–5.6)	0.08
Four-field three-dimensional conformal radiotherapy	V _20 Gy_ ≤ 20%	100	7 (7)		
	V _20 Gy_ > 20%	42	9 (21.4)	3.3 (1.22–8.8)	0.02
Split arc volumetric-modulated arc therapy	V _20 Gy_ ≤ 20%	21	1 (4.8)		
	V _20 Gy_ > 20%	18	2 (11.1)	2.5 (0.22–27.3)	0.46
All modality	V _20 Gy_ ≤ 20%	121	8 (6.6)		
	V _20 Gy_ > 20%	60	11 (18.3)	3.0 (1.19–7.4)	0.02

**Abbreviations:** V_X Gy_; the percentage of lung volume receiving X Gy.

**Table 5:** Univariate analysis of dose–volume histogram and conformity related to radiotherapy techniques

	Radiotherapy technique	Number of patients	Incidence of radiation pneumonitis		Lung						Heart				Conformity index		Homogeneity index	
					V_5 Gy_		V_20 Gy_		Mean dose		V_30 Gy_		Mean dose					
			Rate	*p*- value	Median percentage	*p*- value	Median percentage	*p*- value	Median	*p*- value	Median percentage	*p*- value	Median	*p*- value	Median	*p*- value	Median	*p*- value
Lan, K., *et al.* [11]	3DCRT	297	23.1%^†^	<0.001	75.5%	<0.001	27.2%	0.51	15.8 Gy	<0.001	55.1%	0.003	29.1 Gy	0.27	N/A	N/A	N/A	N/A
	IMRT	91	5.4%^†^		67.5%		27.2%		14.6 Gy		41.1%		27.9 Gy		N/A		N/A	
Kumar, G., *et al.* [14]	3DCRT	23	74.0%^*‡^	0.01	54.4%^*^	0.36	19.5%^*^	0.02	N/A	N/A	N/A	N/A	N/A	N/A	N/A	N/A	1.2^*||^	0.002
	IMRT	22	41.0%^*‡^		59.8%^*^		24.9%^*^		N/A		N/A		N/A		N/A		1.1^*||^	
Munch, S., *et al.* [15]	3DCRT	20	0%^‡^	N/A	79.7%	0.01	21.3%	0.48	N/A	N/A	50.4%	0.02	25.9 Gy	0.15	N/A	N/A	N/A	N/A
	IMRT	17	0%^‡^		90.1%		19.5%		N/A		17.7%		20.6 Gy		N/A		N/A	
Current study	3DCRT	142	11.3%^†^	0.65	40.9%	<0.001	16.5%	0.01	9.1 Gy	<0.001	56.5%	<0.001	30.3 Gy	0.02	2.7^§^	<0.001	0.1^¶^	0.19
	IMRT	39	7.7%^†^		54.4%		19.5%		10.9 Gy		40.8%		28.8 Gy		1.3^§^		0.13^¶^	

**Footnote:**  ^*^ Mean values were described. ^†^RP developed within 12 months after radiotherapy. ^‡^Symptomatic pneumonia that developed during follow-up after radiation therapy.

**Abbreviations:**  ^§^The volume receiving at least 95% dose/the volume of the PTV. ^||^The dose receiving 5% of the CTV/the dose receiving 95% of the CTV. ^¶^(The dose receiving 2% of the PTV − the dose receiving 98% of PTV)/the dose receiving 50% of the PTV. N/A, not available. 3DCRT, three-dimensional conformal radiotherapy. IMRT, intensity-modulated radiotherapy.

## DISCUSSION

The lung volume receiving low-dose irradiation in 4F-3DCRT and SA-VMAT were found to be smaller in our institution compared with the findings of previous studies. Our retrospective report revealed that more conformal doses were delivered to the PTV, sparing heart in patients treated with SA-VMAT compared with that in patients treated with 4F-3DCRT. The lung volumes receiving low to middle doses irradiation were greater in patients treated with SA-VMAT than in patients treated with 4F-3DCRT. However, the increase of the lung volume receiving low-dose irradiation may not always be associated with RP. Furthermore, the presence of ILD was associated with ≥grade 2 RP.

The irradiated lung volume in the current study was smaller than those in the previous studies of the relationship between radiotherapy techniques for thoracic esophageal cancer [11, 14, 15]. For example, V_5 Gy_ of lungs in previous studies was 54.4–79.7% in 3DCRT and 59.8–90.1% in IMRT, while in current study 40.9% and 54.4%, respectively (Table 5). The small CTV margin was probably associated with lower irradiated lung volumes in the current study. Previous studies have added a 3- to 5-cm CTV margin craniocaudally, and a 0.5- to 2-cm CTV margin in the radial direction to the esophageal primary tumor [11, 14, 15]. Sparing the spinal cord was difficult with oblique irradiation fields when the radial CTV margin of the primary esophageal tumor was large. The box-four fields may be required to spare the spinal cord because they encompass larger lung volumes, thus allowing for greater irradiation volume. The current study adopted a 2-cm craniocaudal CTV margin along with the esophagus and a 0- to 0.5-cm CTV margin in the radial direction in accordance with the Japanese Society for Radiation Oncology Guidelines 2020 for Radiotherapy Treatment planning [24]. The current small CTV margin was adopted by the multi-institutional phase II/III trials [22, 25, 26], and this margin did not compromise local–regional control [22, 25, 26]. This approach of small CTV margin helped us to reduce the irradiated lung volumes and may have also played a prominent role in reducing the incidence rate of RP. Target delineation and beam arrangements were found to influence the dose–volume metrics and the respective clinical outcomes, which are necessary to be described and which previous studies have failed to show. The current dose–volume data with distinct planning details would be beneficial to radiotherapy planning for thoracic esophageal cancer.

The dose–volume thresholds for RP in 4F-3DCRT might not be applicable to the thresholds in SA-VMAT. Two differences existed between 4F-3DCRT and SA-VAMT. First, the effect of maintaining a continuous and uniform dose delivery in 4F-3DCRT may not be applicable to heterogeneous dose accumulations of small irradiation fields in SA-VMAT. The cell surviving fraction tended to increase in groups receiving a larger dose of fractionated irradiation compared with continuous or small fractionated irradiation [27]. Second, our findings showed that SA-VMAT could deliver more conformal doses to the target compared with 4F-3DCRT. Conformal dose delivery using SA-VMAT reduced the irradiated dose to the heart and the surrounding organs, which might also indirectly reduce RP, despite the increased lung volume receiving low to middle doses irradiation. Experimental studies using rats reported the association between cardiac irradiation and radiation-induced pulmonary injury [28]. Retrospective studies have also reported that the increase of irradiation doses to heart and to pulmonary artery are significant risk factors for the development of RP in mesothelioma and thymic tumors [29, 30]. A secondary analysis of the randomized trial of non-small cell lung cancer reported that V_5 Gy_ of lungs was larger, and a lower cardiac dose was found in IMRT compared with 3DCRT, but the incidence of RP was lower in IMRT [31]. Conformal dose delivery of SA-VMAT managed to spare heart and major arteries in radiotherapy for thoracic esophageal cancer, which might counteract the increased the lung volume receiving low to middle doses irradiation. The characteristics of dose delivery and conformal dose delivery of SA-VAMT may not always increase the incidence of RP, despite increased the lung volume receiving low to middle doses irradiation.

Finally, our multivariate analysis indicated that the presence of ILD was one of the risk factors for the development of RP in esophageal cancer. Our results correspond with the findings of previous studies for lung cancer [20]. In our study, all patients with ILD had no respiratory symptoms, and six out of seven patients were first diagnosed with ILD at the time of diagnosis for esophageal cancer. However, three out of seven patients eventually developed ≥grade 2 RP. Therefore, it is important to remember that patients with esophageal cancer may suffer from undiagnosed ILD. Screening for asymptomatic ILD before radiotherapy for esophageal cancer is useful to specify patients with increased risk of RP and perform shared decision-making processes with patients.

The current study has several limitations. The number of patients included was relatively small, and thus, the power of this study may not be enough to detect small differences among patients. In contrast, the excluded number of patients from this cohort was significantly large, which might have resulted in selection bias. We identified several confounders: SA-VMAT was frequently used for upper thoracic esophageal cancer, advanced stage and for large PTV volumes. Those bias might influence the different incidence rate of RP between 4F-3DCRT and SA-VAMT; however we tried to adjust these cofounders so as to reduce their impact on our study. Information bias might also have influenced the number of events and the severity of RP, although we tried to clearly define RP. Despite these limitations, the current study is the first to report that SA-VMAT increased the lung volume receiving low to middle doses irradiation but might not always increase RP while showing clear study setting, inclusion criteria, exposure (radiotherapy details) and the definition of events compared with previous studies [11, 14–17]. The current results might help radiation oncologists to select SA-VMAT for thoracic esophageal cancer with advanced stage or large PTV at least which the delivery of adequate doses to targets by would be difficult in 4F-3DCRT.

In conclusion, our retrospective cohort showed that the lung volume receiving low to middle doses irradiation was larger in patients treated with SA-VMAT compared with that of patients treated with 4F-3DCRT. However, this increase may not always be associated with RP. Further studies are needed to explore the effect of conformal dose of SA-VMAT on RP.

## Supplementary Material

Supplementary_Table_1_rrac021
