# Supplementary material for: Association of volumetric-modulated arc therapy with radiation pneumonitis in thoracic esophageal cancer
Source: J Radiat Res. 2022 May 20;63(4):646–56. doi: 10.1093/jrr/rrac021 (PMC9303599; doi:10.1093/jrr/rrac021)
Supplement: Supplementary_Table_1_rrac021 [file supplementary_table_1_rrac021.docx]

**Supplementary Table 1. Details of prescribed dose**

|  | Prescribed dose (Gy) | Number of patients |
| --- | --- | --- |
| Four-field three-dimensional conformal radiotherapy | 50.4 | 38 |
|  | 54 | 2 |
|  | 56 | 1 |
|  | 58 | 1 |
|  | 59.4 | 2 |
|  | 59.8 | 1 |
|  | 60 | 95 |
|  | 61 | 1 |
|  | 66 | 1 |
| Split arc volumetric-modulated arc therapy | 50.4 | 13 |
|  | 60 | 13 |
|  | 60.4 | 1 |
|  | 66 | 11 |
|  | 69 | 1 |
